# CoMS-UNet: A Cohesive Multi-Scale Network for Semantic Reorganization and Scale-Aware Context Modeling in Remote Sensing Image Segmentation

**DOI:** 10.3390/s26154987

**Published:** 2026-08-06

**Authors:** Yankai Wang, Shaochen Jiang, Liejun Wang, Beibei Gao

**Affiliations:** School of Computer Science and Technology, Xinjiang University, Huarui Street, Urumqi 830046, China; 107552503687@stu.xju.edu.cn (Y.W.); 107552403898@stu.xju.edu.cn (B.G.)

**Keywords:** remote sensing semantic segmentation, high-resolution remote sensing imagery, multi-scale semantic reorganization, scale-aware learning, land-cover interpretation

## Abstract

Semantic segmentation is essential for fine-grained land-cover interpretation from high-resolution remote sensing imagery. However, existing methods still show limitations in coordinating multi-stage encoder features, providing scale-aware contextual guidance at the bottleneck layer, and facilitating cross-channel information exchange during decoder reconstruction. To address these issues, this paper proposes CoMS-UNet, a cohesive multi-scale U-Net that integrates semantic reorganization, scale-aware context modeling, and channel-aware decoder reconstruction. In encoder–decoder skip connections, the Multi-Scale Semantic Reorganization Module (MSRM) reorganizes multi-stage encoder features through a fusion–separation–refusion process to achieve cross-scale semantic alignment. At the bottleneck stage, the Scale-Aware Module (SAM) constructs predefined receptive-field branches and adaptively weights their contextual responses to enhance scale-relevant semantic guidance. During decoder reconstruction, the Channel-Spatial Shuffle Mamba Block (CSSMBlock) promotes cross-channel information exchange and long-range spatial dependency modeling to improve structural detail recovery. Through this unified feature-processing framework, CoMS-UNet strengthens semantic consistency, scale-aware context aggregation, and channel-aware decoder reconstruction. Experiments on the ISPRS Vaihingen dataset and the labeled LoveDA validation set show that CoMS-UNet achieves a mean mIoU of 86.11±0.35% over three independent runs on Vaihingen and an mIoU of 52.48% on LoveDA.

## 1. Introduction

Remote sensing semantic segmentation aims to assign a semantic category to each pixel in a remote sensing image, thereby enabling detailed interpretation of land cover and target distribution. With the increasing availability of high-resolution Earth observation imagery, remote sensing semantic segmentation has become increasingly important in crop classification [1], agricultural parcel delineation [2], coastal wetland monitoring [3], and post-disaster damage assessment [4]. Compared with conventional computer vision datasets, high-resolution remote sensing datasets generally contain more complex backgrounds and greater object-scale variation. Existing semantic segmentation methods, especially convolutional encoder–decoder architectures, have improved pixel-wise segmentation accuracy by extracting hierarchical features and using decoder structures to recover spatial details. However, remote sensing datasets typically exhibit complex spatial layouts, significant object-scale variations, spectral differences, and blurred boundaries. These factors may lead to fragmented land-cover boundaries, confusion between visually similar categories, and incomplete extraction of small or narrow objects in fine-grained land-cover interpretation. Therefore, improving the model’s ability to maintain semantic consistency, adapt to scale variations, and recover fine structural details remains essential for reliable interpretation of high-resolution remote sensing imagery.

Convolutional Neural Networks (CNNs) [5,6] have laid an important foundation for semantic segmentation because they can extract hierarchical visual representations from images. On this basis, Fully Convolutional Networks (FCNs) [7] perform dense pixel-wise prediction by replacing the fully connected layers with convolutional operations, while U-Net [8] builds an encoder–decoder architecture and uses skip connections to recover spatial details, making it widely adopted in segmentation tasks. Attention U-Net [9] further enhances the skip pathways by introducing attention gates, which filter encoder features before their fusion with decoder features. In addition to these encoder–decoder designs, multi-scale contextual modeling has been widely studied. DeepLabV3+ [10] relies on Atrous Spatial Pyramid Pooling (ASPP), whereas PSPNet [11] adopts Pyramid Pooling Module (PPM), allowing high-level contextual features to be captured from different receptive-field ranges. Depthwise Separable Convolution (DSC) improves computational efficiency by decoupling spatial filtering and channel mixing, and ShuffleNet [12] uses channel shuffle to promote information exchange among channels. With the emergence of state-space models and the Mamba architecture, CM-UNet [13] integrates CNN-based local feature extraction, Mamba-based long-range dependency modeling, and a Multi-Scale Attention Aggregation (MSAA) module to fuse multi-scale encoder features for remote sensing segmentation.

Against this background, recent studies have further explored boundary-aware multi-scale representation and state-space modeling for remote sensing image segmentation. BEMS-UNetFormer [14] emphasizes boundary enhancement and multi-scale context aggregation, whereas MLMFPN [15] focuses on local–global synergy and cross-scale context interaction. CVMH-UNet [16], PyramidMamba [17], and SwinMamba [18] further combine state-space modeling with multi-scale multi-frequency fusion, pyramid feature fusion, and hybrid local–global modeling, respectively. These advances highlight the continuing importance of multi-scale representation, local detail modeling, and long-range dependency modeling in high-resolution remote sensing image segmentation.

Despite these advances, several challenges remain in fine-grained land-cover interpretation from high-resolution remote sensing imagery: (1) Multi-stage encoder features differ in spatial detail and semantic abstraction, and insufficient coordination may cause fragmented predictions. (2) Fixed bottleneck aggregation may not adequately adapt to substantial object-scale variation. (3) Limited inter-channel interaction during decoding may weaken boundary and structural detail reconstruction.

The main contributions of this paper are summarized as follows:

- We propose the Multi-Scale Semantic Reorganization Module (MSRM), which strengthens cross-scale semantic connections and promotes semantic reorganization of multi-stage encoder features in skip connections. By adopting a fusion–separation–refusion strategy, MSRM aligns, fuses, and reassembles hierarchical encoder features, thereby alleviating the interference caused by noisy shallow spatial details and the spatial detail degradation of deep contextual representations in high-resolution remote sensing scenes.
- We design a Scale-Aware Module (SAM) to improve the model’s sensitivity to target scale variations in remote sensing imagery. SAM constructs receptive-field branches with predefined dilation rates and adaptively weights their contextual responses according to the input feature. By learning input-dependent branch weights at the bottleneck layer, SAM selectively emphasizes scale-relevant contextual cues, thereby providing semantic guidance for decoder reconstruction.
- We propose the Channel-Spatial Shuffle Mamba Block (CSSMBlock) to strengthen channel-aware decoder reconstruction, thereby facilitating the effective recovery of fine structural details in remote sensing imagery. CSSMBlock integrates channel shuffle-based local extraction, SS2D-based long-range modeling, and dual attention to alleviate the channel-wise information isolation caused by depthwise separable convolution. By promoting cross-channel joint modeling during decoder reconstruction, CSSMBlock improves the recovery of object boundaries, narrow structures, and fragmented land-cover regions.

## 2. Materials and Methods

### 2.1. Architecture Overview

**Figure 1:**
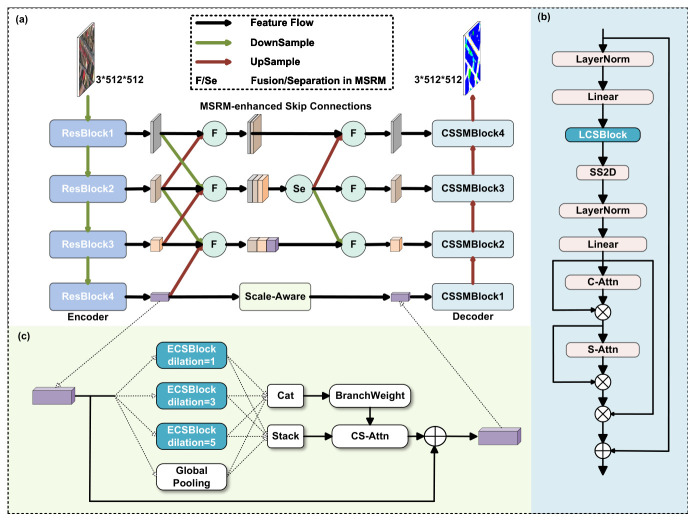
Overall architecture of CoMS-UNet. (**a**) CoMS-UNet. (**b**) CSSMBlock. (**c**) Scale-Aware Module. F and Se indicate the fusion and separation operations in MSRM, respectively. C-Attn, S-Attn, and CS-Attn denote channel, spatial, and channel-spatial attention, respectively. The Enhanced Channel-Shuffle Block (ECSBlock) is used to construct multi-scale receptive-field branches in SAM, while the Local Channel-Shuffle Block (LCSBlock) is used for local feature extraction in CSSMBlock.

**Figure 2:**
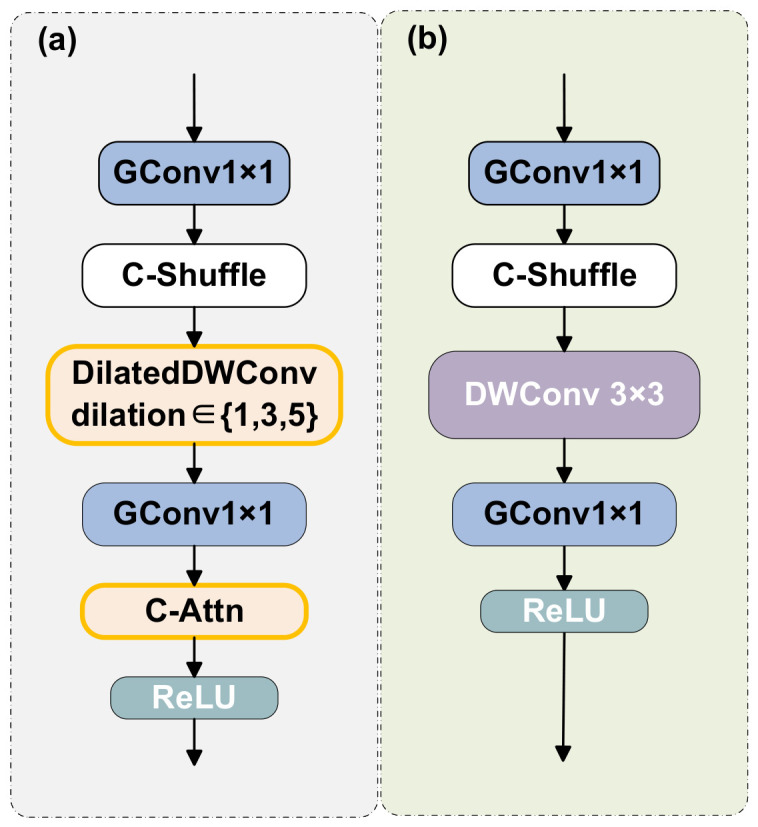
Architectural details of the proposed feature blocks. (**a**) Enhanced Channel-Shuffle Block (ECSBlock). (**b**) Local Channel-Shuffle Block (LCSBlock). Three ECSBlock instances with dilation rates of 1, 3, and 5 are used to construct the receptive-field branches in SAM. LCSBlock is used in the decoder and adopts a standard 3×3 depthwise convolution. GConv1 × 1, C-Shuffle, DWConv, and C-Attn denote grouped 1×1 convolution, channel shuffle, depthwise convolution, and channel attention, respectively.

The proposed CoMS-UNet (Figure 1) follows an encoder–bottleneck–decoder structure and forms a cohesive feature-processing framework through multi-scale semantic alignment, adaptive bottleneck aggregation, and channel-aware decoder reconstruction. To improve the semantic coordination of multi-stage encoder features in skip connections, MSRM introduces learnable cross-level interaction instead of direct feature stacking. This design promotes the complementary integration of shallow spatial details and deep contextual representations, thereby strengthening cross-scale semantic connections and improving the semantic organization of multi-scale features. We use the Enhanced Channel-Shuffle Block (ECSBlock) and the Local Channel-Shuffle Block (LCSBlock) in SAM and CSSMBlock, respectively. The specific structures of ECSBlock and LCSBlock are shown in Figure 2a and Figure 2b, respectively. SAM addresses the fixed aggregation pattern at the bottleneck stage by constructing ECSBlock-based receptive-field branches with predefined dilation rates and adaptively weighting their contextual responses according to the input feature. As for the decoder, our proposed CSSMBlock internally replaces the standard DSC with LCSBlock. Through the channel shuffle operation, LCSBlock facilitates cross-channel information exchange, alleviates the channel-wise information isolation caused by depthwise convolution, and helps maintain feature coherence during decoder reconstruction.

### 2.2. Multi-Scale Semantic Reorganization Module (MSRM)

CoMS-UNet introduces a Multi-Scale Semantic Reorganization Module (MSRM) to improve cross-scale semantic coordination in skip connections. MSRM reorganizes hierarchical encoder features through a fusion-separation–refusion process. This design strengthens cross-scale semantic connections and enables more coherent integration of shallow spatial details and deep contextual representations.

Given the hierarchical encoder features ${\left\{E_{i}\right\}}_{i=1}^{4}$, MSRM first aligns neighboring features to the target scale using 1×1 channel projection and bilinear interpolation. Let ${\tilde{E}}_{j\to i}$ denote the feature from level *j* after being aligned to level *i*. The preliminary fusion stage aggregates complementary contextual information from adjacent levels through channel-wise concatenation:

(1) $$\begin{array}{cc} F_{1} & =F_{1}\left([E_{1},{\tilde{E}}_{2\to 1}]\right), \end{array}$$

(2) $$\begin{array}{cc} F_{2} & =F_{2}\left([{\tilde{E}}_{1\to 2},E_{2},{\tilde{E}}_{3\to 2}]\right), \end{array}$$

(3) $$\begin{array}{cc} F_{3} & =F_{3}\left([{\tilde{E}}_{2\to 3},E_{3},{\tilde{E}}_{4\to 3}]\right), \end{array}$$

The fusion function $F_{i}$ consists of two stacked 3×3 convolutions, batch normalization, ReLU activation, and a channel attention mechanism that dynamically recalibrates channel weights via global average pooling and a two-layer fully connected bottleneck.

To obtain a balanced basis for subsequent feature separation, $F_{2}$ is used as the central hub between semantic abstraction and spatial detail preservation. From this unified central hub, we apply three parallel separation branches to decouple scale-specific feature representations:

(4) $$\begin{array}{cc} S_{1} & =S_{1}\left(F_{2}\right), \end{array}$$

(5) $$\begin{array}{cc} S_{2} & =S_{2}\left(F_{2}\right), \end{array}$$

(6) $$\begin{array}{cc} S_{3} & =S_{3}\left(F_{2}\right), \end{array}$$

Each of the separation functions $S_{i}$ is implemented through a 3×3 convolution, batch normalization, ReLU, and the aforementioned channel attention mechanism. After these operations, we perform bilinear interpolation to resize the feature map to match the corresponding target resolution.

In the final fusion stage after separation, these separated features are recombined with the corresponding scaled features from the initial fusion. This integration incorporates multi-scale contextual information, enabling each level of feature to simultaneously possess high-level semantic information, while also retaining the spatial information of the shallow features:

(7) $${\hat{E}}_{1}=R_{1}\left([F_{1},{\tilde{S}}_{1}]\right),$$

(8) $${\hat{E}}_{2}=S_{2},$$

(9) $${\hat{E}}_{3}=R_{3}\left([F_{3},{\tilde{S}}_{3}]\right),$$

${\tilde{S}}_{i}$ represents the separated features after being aligned to the corresponding scale. The refinement function $R_{i}$ has the same configuration structure as $F_{i}$. Then, the obtained $\left\{{\hat{E}}_{1},{\hat{E}}_{2},{\hat{E}}_{3},E_{4}\right\}$ is forwarded to the decoder.

### 2.3. Scale-Aware Module (SAM)

The Scale-Aware Module (SAM) is introduced at the bottleneck stage to enhance the scale-aware representation of deep features and provide more adaptive contextual guidance for decoder reconstruction. In high-resolution remote sensing scenes, fixed aggregation patterns may limit the ability of bottleneck features to emphasize scale-relevant contextual cues. To address this issue, SAM constructs multiple ECSBlock-based receptive-field branches and adaptively weights their contextual responses according to the input feature. Specifically, ECSBlock consists of a grouped 1×1 convolution, a channel shuffle operation for inter-group information exchange, a depthwise dilated convolution (${\mathrm{DWConv}}_{3\times 3}^{d}$) for spatial context modeling, a final grouped 1×1 convolution, and a channel attention mechanism for channel-wise recalibration. This structure refines each branch feature before multi-branch aggregation.

Based on this structure, SAM adopts four parallel paths. The first three paths employ ECSBlocks with predefined dilation rates d∈{1,3,5}, while the fourth path introduces image-level contextual information through global average pooling (GAP). Let $H_{d}$ denote the ECSBlock with dilation rate *d*, and let X denote the bottleneck input feature:

(10) $$B_{1}=H_{1}\left(X\right),\;B_{2}=H_{3}\left(X\right),\;B_{3}=H_{5}\left(X\right),$$

With a 3×3 depthwise dilated convolution, the three branches correspond to effective receptive fields of 3×3, 7×7, and 11×11, respectively. These branches allow the bottleneck feature to capture contextual responses from different spatial ranges, including fine local details, medium-range patterns, and larger-range contextual cues. To further complement these receptive-field branches, the fourth branch introduces image-level contextual information through global average pooling (GAP):

(11) $$B_{4}=U\left(\delta (\mathrm{BN}\left({\mathrm{Conv}}_{1\times 1}\left(\mathrm{GAP}\left(X\right)\right)\right))\right),$$

where U denotes bilinear upsampling, BN is batch normalization, and δ is the ReLU activation.

Rather than simply concatenating or summing the branch outputs, SAM explicitly models the relative importance of different scale responses through an input-dependent branch weighting mechanism. The concatenated multi-branch feature is denoted as $B_{\mathrm{cat}}=\left[B_{1},B_{2},B_{3},B_{4}\right]$. It is then used to generate adaptive branch weights $\alpha \in R^{4}$:

(12) $$\alpha =\mathrm{Softmax}\left({\mathrm{Conv}}_{1\times 1}\left(\mathrm{GAP}\left(\delta \left(\mathrm{BN}\left({\mathrm{Conv}}_{1\times 1}\left(B_{\mathrm{cat}}\right)\right)\right)\right)\right)\right).$$

The branch-weighted feature is obtained by aggregating the contextual responses from the four paths:

(13) $$F=\sum_{i=1}^{4}\alpha_{i}B_{i}.$$

The aggregated feature is further refined by a joint channel-spatial attention module, denoted as $A_{\mathrm{cs}}$. Finally, the attended feature is projected by a 1×1 convolution and combined with the original bottleneck input through a residual connection:

(14) $$\hat{O}=\delta \left(\mathrm{BN}({\mathrm{Conv}}_{1\times 1}\left(A_{\mathrm{cs}}\left(F\right)\right))\right)+X.$$

Through predefined receptive-field branches, input-dependent branch weighting, channel-spatial refinement, and residual learning, SAM enables bottleneck features to emphasize scale-relevant contextual cues. This process improves the scale-aware semantic guidance delivered from the bottleneck layer to the decoder.

### 2.4. Channel-Spatial Shuffle Mamba Block (CSSMBlock)

CSSMBlock is designed to enhance channel-aware decoder reconstruction by promoting inter-channel information exchange and structural detail recovery. To this end, it follows a sequential refinement process consisting of LCSBlock-based local modeling, SS2D-based long-range dependency modeling, and channel-spatial attention refinement.

Given an input feature map $X\in R^{C\times H\times W}$, CSSMBlock first reshapes it into a sequence representation $X_{seq}$. Then, layer normalization and linear projection are applied to expand the channel dimension:

(15) $$Z_{1}={\mathrm{Linear}}_{\mathrm{up}}\left(\mathrm{LN}\left(X_{\mathrm{seq}}\right)\right),$$

where $Z_{1}$ has an expanded channel dimension with an expansion factor λ=2.

The expanded sequence is reshaped back into a spatial feature map and then processed by LCSBlock for local feature extraction, where $H_{\mathrm{LCS}}$ denotes the LCSBlock transformation:

(16) $$Z_{2}=H_{\mathrm{LCS}}\left(\mathrm{Reshape}\left(Z_{1}\right)\right).$$

LCSBlock employs grouped 1×1 convolution and channel shuffle to facilitate information exchange across channel groups. Different from the ECSBlock used in the bottleneck stage, LCSBlock adopts a standard non-dilated 3×3 depthwise convolution and does not include an internal channel attention mechanism. This design focuses on local structural modeling while alleviating the channel-wise information isolation caused by the depthwise convolution in DSC.

To further model long-range spatial dependencies beyond local convolutional extraction, the locally encoded feature is processed by the two-dimensional state space model, denoted as SS2D:

(17) $$Z_{3}=M_{\mathrm{SS}2D}\left(Z_{2}\right).$$

Through two-dimensional selective scanning, SS2D models spatial dependencies across distant regions and enriches the structural context required for decoder reconstruction. The resulting feature is then normalized and projected back to the original channel dimension:

(18) $$Z_{4}={\mathrm{Linear}}_{\mathrm{down}}\left(\mathrm{LN}\left(Z_{3}\right)\right).$$

After the channel projection, $Z_{4}$ is reshaped back to the spatial form for the subsequent channel-spatial attention refinement.

CSSMBlock further introduces a dual attention mechanism to refine decoder features from both channel and spatial dimensions. The attention-refined features are formulated as

(19) $$\begin{array}{cc} Z_{c} & =A_{c}\left(Z_{4}\right)⊙Z_{4}, \end{array}$$

(20) $$\begin{array}{cc} Z_{\mathrm{cs}} & =A_{s}\left(Z_{c}\right)⊙Z_{c}, \end{array}$$

(21) $$\begin{array}{cc} Z_{f} & =Z_{4}⊙Z_{\mathrm{cs}}, \end{array}$$

where $A_{c}$ and $A_{s}$ denote the channel attention and spatial attention operations, respectively, $Z_{f}$ denotes the fused attention-refined feature, and ⊙ represents element-wise multiplication.

Finally, a residual connection is introduced to preserve the original feature information and stabilize decoder feature reconstruction:

(22) $$Y=X+Z_{f}.$$

Through the progressive refinement from local structural modeling to long-range dependency modeling and channel-spatial attention, CSSMBlock strengthens cross-channel joint modeling during decoder reconstruction. This improves the recovery of object boundaries and fine structural details.

## 3. Results

### 3.1. Experimental Setup

#### 3.1.1. Dataset Settings

**ISPRS Vaihingen Dataset.** The ISPRS Vaihingen benchmark includes 33 high-resolution aerial images acquired over Vaihingen, Germany, with a ground sampling distance of 9 cm. In our experiments, 16 images are assigned to the training set, and the other 17 images are used for testing.

**LoveDA Dataset.** LoveDA is a high-spatial-resolution remote sensing benchmark for land-cover semantic segmentation and unsupervised domain adaptation. We use the official training split, comprising 2522 images, for model training and the labeled validation split, comprising 1669 images, for evaluation. Since the labels of the official test set are not publicly available, all LoveDA results are reported on the labeled validation set.

#### 3.1.2. Implementation Details

The experiments were implemented in PyTorch 2.0 and conducted on a single NVIDIA A40 GPU (NVIDIA Corporation, Santa Clara, CA, USA). For CoMS-UNet, the encoder was initialized with pretrained ResNet-18 weights. The model was trained for 105 epochs with a batch size of 8. AdamW combined with Lookahead was used for optimization. The learning rate was annealed from $6\times 10^{-4}$ to $1\times 10^{-5}$ using a cosine schedule, and the weight decay was set to 0.01. The training objective combined soft cross-entropy loss, Dice loss, and auxiliary supervision from three decoder stages. Each auxiliary loss was assigned a weight of 0.4.

Training samples were generated by random scaling followed by content-balanced cropping into 512-by-512-pixel patches. The maximum proportion of the dominant class within a crop was set to 0.75. Scale factors of 0.5, 0.75, 1.0, 1.25, and 1.5 were used for ISPRS Vaihingen. For LoveDA, the corresponding factors were 0.75, 1.0, 1.25, and 1.5. Four-image mosaic augmentation was applied to both datasets with a probability of 0.25. Random 90-degree rotation was applied to ISPRS Vaihingen, whereas random horizontal flipping was applied to LoveDA. Each dataset-specific transformation was used with a probability of 0.5.

For CoMS-UNet, inference on the ISPRS Vaihingen dataset was performed using offline-generated 1024-by-1024-pixel tiles. LoveDA images were evaluated directly at their native 1024-by-1024-pixel resolution. Each input was processed as a whole, without additional online sliding-window partitioning. Multi-scale and flip-based test-time augmentation was applied, and the resulting prediction scores were averaged to obtain the final output.

#### 3.1.3. Evaluation Metrics and Comparison Protocol

To compare CoMS-UNet with existing methods, the mean F1 score (mF1), mean Intersection over Union (mIoU), and overall accuracy (OA) are used as evaluation metrics. Among them, mF1 reflects the balance of precision and recall across different classes, mIoU more strictly measures the overlap between the predicted labels and the true annotations, and OA evaluates the pixel-wise classification accuracy of the model. By comprehensively considering these three metrics, the performance of CoMS-UNet can be evaluated from the category level and the global level. For the ISPRS Vaihingen dataset, the reported scores were computed using eroded reference boundaries, and boundary pixels were ignored. The clutter class was included in the confusion matrix and the calculation of overall accuracy (OA). Its individual IoU and F1 scores were computed but are not reported in Table 1, and it was excluded from the calculation of mIoU and mean F1.

All comparative methods reported in Table 1 and Table 2 were reproduced in our experimental environment. Model-specific implementation settings, including training hyperparameters, input preprocessing, inference procedures, and test-time augmentation, were based on the corresponding publications or official implementations. Within each dataset, all methods used the same data split, evaluation images, reference labels, and metric implementation. The citations accompanying the method names refer to the corresponding original publications.

The main comparison results were obtained from runs using random seed 42. To examine run-to-run variation, each configuration in the ablation study was independently trained using random seeds 0, 1, and 42. Results from these runs are reported as the mean ± standard deviation.

### 3.2. Results on ISPRS Vaihingen Dataset

Table 1 presents the quantitative results on the ISPRS Vaihingen dataset. CoMS-UNet obtains an mF1 of 92.58%, an OA of 95.01%, and an mIoU of 86.51%. Compared with the CM-UNet baseline, CoMS-UNet improves mF1, OA, and mIoU by 0.77, 0.52, and 1.23 percentage points, respectively. Improvements are also observed in the F1 scores of all five land-cover classes.

**Figure 3:**
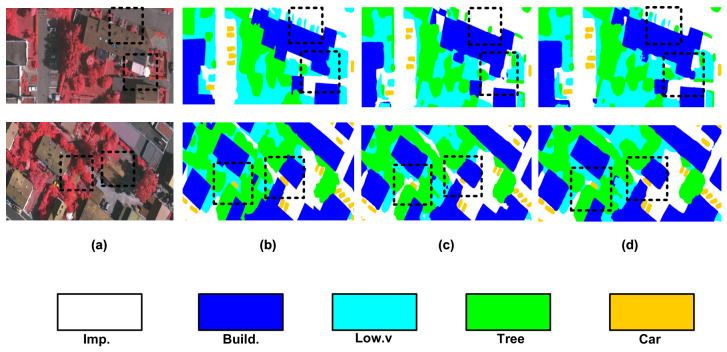
Visual comparisons on the ISPRS Vaihingen dataset. Columns (**a**–**d**) show RGB images, ground truth, CM-UNet, and CoMS-UNet (Ours), respectively.

Figure 3 presents a visual comparison between CM-UNet and CoMS-UNet on the ISPRS Vaihingen dataset. Compared with CM-UNet, CoMS-UNet produces more continuous segmentation regions and preserves object boundaries and small targets more completely in several examples. Among the class-wise results, the F1 scores for Low vegetation, Tree, and Car increase by 1.91, 0.69, and 0.68 percentage points, respectively. These results indicate that CoMS-UNet reduces some fragmented predictions and improves the segmentation of vegetation regions and small objects.

### 3.3. Results on LoveDA Dataset

Table 2 presents the quantitative results on the labeled LoveDA validation set. CoMS-UNet obtains an mIoU of 52.48%, which is 1.01 percentage points higher than that of the CM-UNet baseline. The results indicate that the modifications introduced in CoMS-UNet provide an improvement over the baseline on the LoveDA dataset.

**Figure 4:**
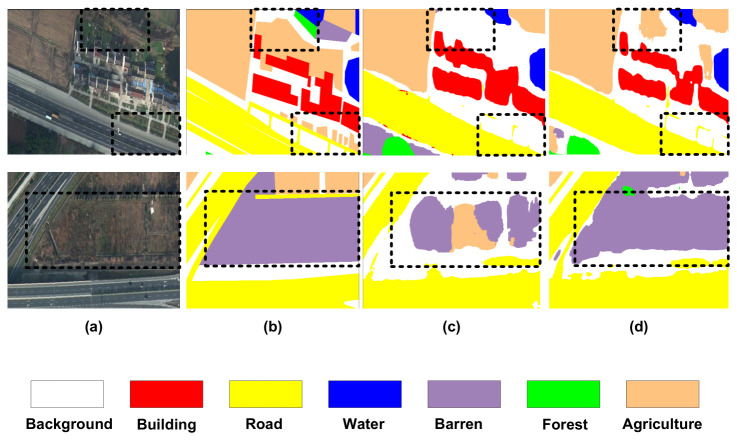
Results on the labeled LoveDA validation set. Columns (**a**–**d**) show RGB images, ground truth, CM-UNet, and CoMS-UNet (Ours), respectively.

Figure 4 presents a visual comparison between CM-UNet and CoMS-UNet on the labeled LoveDA validation set. Compared with CM-UNet, CoMS-UNet produces more coherent segmentation regions in several examples and better preserves some land-cover boundaries. Among the class-wise results, the IoU scores for Forest, Water, Building, Background, and Agriculture increase by 2.72, 1.57, 0.93, 0.90, and 0.84 percentage points, respectively. In contrast, the IoU for Road decreases slightly by 0.26 percentage points. These results indicate that CoMS-UNet improves the segmentation of several land-cover categories, although the improvement is not uniform across all classes.

### 3.4. Ablation Study

Table 3 presents the ablation results on the ISPRS Vaihingen dataset. Compared with the baseline mIoU of 84.88±0.41%, the MSRM-only, SAM-only, and CSSMBlock-only variants achieve mean mIoU scores of 85.93±0.19%, 85.49±0.60%, and 85.26±0.40%, respectively. Among the single-module configurations, MSRM provides the largest mean mIoU increase. The complete CoMS-UNet achieves the highest mean mF1 and mIoU, reaching 92.33±0.22% and 86.11±0.35%, respectively, with a mean mIoU improvement of 1.23 percentage points over the baseline. Although the MSRM-only variant obtains a slightly higher mean OA than the complete model, the difference is only 0.03 percentage points and is small relative to the observed run-to-run variation. Therefore, the two configurations show similar OA results.

Among the two-module configurations, MSRM+SAM achieves the highest mean mIoU of 86.05±0.12%, followed by MSRM+CSSMBlock with 85.84±0.02% and SAM+CSSMBlock with 85.48±0.31%. The complete CoMS-UNet obtains a mean mIoU of 86.11±0.35%. These results show that combining the modules does not produce strictly additive improvements, and the performance differences among several module combinations are relatively small.

**Figure 5:**
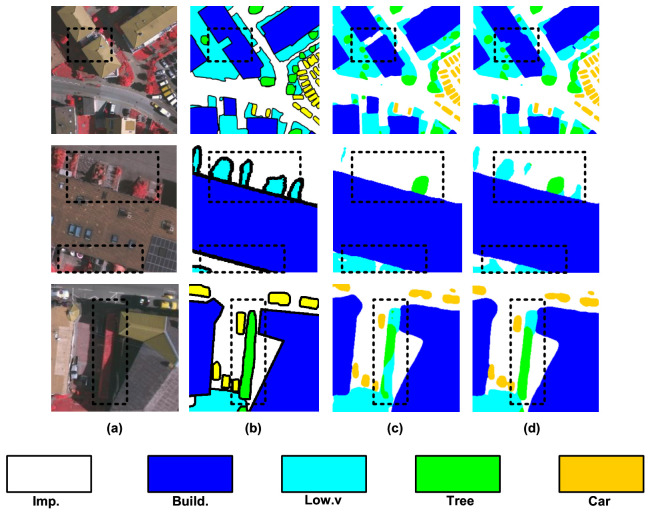
Qualitative ablation results on the ISPRS Vaihingen dataset. The three rows show the visualization results of the MSRM-only, SAM-only, and CSSMBlock-only variants, respectively. Columns (**a**–**d**) show the RGB image, ground truth, CM-UNet baseline, and the corresponding single-module result, respectively.

To further demonstrate the effects of the proposed modules, Figure 5 presents qualitative ablation results on representative challenging regions from the ISPRS Vaihingen dataset. In the first row, the baseline produces fragmented predictions and incoherent object structures around buildings, whereas MSRM helps improve the semantic consistency of multi-stage encoder features and generate more coherent land-cover structures. In the second row, SAM recovers regions with different spatial sizes more completely, suggesting that it helps select more appropriate receptive-field responses. In the third row, CSSMBlock improves the reconstruction of visually similar structures, indicating that enhanced inter-channel interaction contributes to more consistent decoder reconstruction. These visual results provide qualitative evidence consistent with the quantitative ablation results in Table 3 and illustrate how the proposed modules alleviate different limitations in semantic coordination, scale-aware context modeling, and decoder reconstruction.

### 3.5. Complexity and Efficiency Analysis

Table 4 compares the computational cost, parameter count, and segmentation accuracy of the evaluated models on the ISPRS Vaihingen dataset. Compared with CM-UNet, the FLOPs of CoMS-UNet increase from 12.02 to 38.48 GFLOPs at 512×512 and from 48.08 to 153.91 GFLOPs at 1024×1024, while the parameter count increases from 12.89 M to 30.86 M. The additional computational cost is associated with the feature reorganization, multi-branch aggregation, and decoder reconstruction operations introduced in CoMS-UNet.

Compared with CM-UNet, CoMS-UNet increases the mIoU from 85.28% to 86.51%, corresponding to an improvement of 1.23 percentage points. However, this improvement is accompanied by approximately 3.2× FLOPs and 2.39× parameters, indicating a substantial increase in computational cost. Compared with CMTFNet, CoMS-UNet has a similar parameter count but requires more FLOPs and obtains a 4.65-percentage-point higher mIoU. Compared with FTUNetFormer, CoMS-UNet uses approximately one-third of its parameters and FLOPs while obtaining a 2.37-percentage-point higher mIoU. Overall, the current architecture places greater emphasis on segmentation accuracy than computational efficiency, and reducing its computational cost and parameter count remains an important direction for future work.

## 4. Discussion

The results across the two datasets suggest that the modifications introduced at the skip connections, bottleneck, and decoder contribute to the segmentation performance of CoMS-UNet. However, the small difference between the complete model and the MSRM+SAM variant indicates that the effects of the modules are not strictly additive. Meanwhile, CoMS-UNet does not introduce a new segmentation paradigm, but modifies different stages of the feature-processing pipeline through semantic reorganization, scale-aware contextual modeling, and channel-aware reconstruction.

CoMS-UNet also has several limitations. Compared with CM-UNet, its FLOPs increase by approximately 3.2× at both input resolutions, while the parameter count increases from 12.89 M to 30.86 M. Therefore, the accuracy improvement is accompanied by a substantial increase in computational cost. Performance also remains uneven across land-cover categories, particularly for narrow and continuous structures. Finally, the experiments are limited to two public datasets and do not fully establish generalization across different sensors, regions, seasons, and spatial resolutions. Future work will therefore focus on reducing model complexity, improving the segmentation of narrow structures and small objects, and evaluating the model under broader cross-domain and multimodal settings.

## 5. Conclusions

In this study, we present CoMS-UNet for remote sensing image semantic segmentation by integrating multi-scale semantic reorganization, scale-aware contextual modeling, and channel-aware decoder reconstruction. MSRM reorganizes multi-stage encoder features, SAM provides scale-aware contextual information at the bottleneck layer, and CSSMBlock enhances inter-channel interaction and spatial dependency modeling during decoder reconstruction. On the ISPRS Vaihingen dataset, CoMS-UNet achieves a mean mIoU of 86.11±0.35% over three independent runs, representing a mean improvement of 1.23 percentage points over the CM-UNet baseline. On the labeled LoveDA validation set, CoMS-UNet obtains an mIoU of 52.48%. These results indicate that the proposed feature-processing design across the skip connections, bottleneck, and decoder can improve segmentation accuracy. However, the improvement is accompanied by increased computational cost and parameter count. Future work will prioritize model simplification and broaden the scope of applications in remote sensing.

## Figures and Tables

**Table 1:** Experimental results (%) on the ISPRS Vaihingen dataset. Class-wise values denote F1 scores, and bold values indicate the results of CoMS-UNet.

Method	Backbone	Imp.	Build.	Low.v	Tree	Car	mF1	OA	mIoU
ABCNet [19]	R18	96.25	94.41	83.96	89.94	81.12	89.14	92.56	80.90
BANet [20]	ResT	96.61	94.88	84.20	89.34	83.35	89.68	92.75	81.72
Segmenter [21]	ViT-T	95.33	93.61	76.31	88.05	58.87	82.43	91.45	72.22
UNetFormer [22]	R18	96.60	95.45	83.44	89.39	86.83	90.34	92.88	82.77
FTUNetFormer [22]	Swin-B	97.01	95.76	84.57	89.99	88.62	91.19	93.39	84.14
CMTFNet [23]	R50	96.13	94.43	83.66	89.20	85.62	89.81	92.33	81.86
RS3Mamba [24]	R18-Mamba	96.77	96.10	81.97	91.11	89.72	91.13	94.05	84.14
CM-UNet [13]	R18	97.14	96.51	82.67	91.73	91.00	91.81	94.49	85.28
**CoMS-UNet (Ours)**	R18	**97.46**	**96.75**	**84.58**	**92.42**	**91.68**	**92.58**	**95.01**	**86.51**

**Table 2:** Experimental results (%) on the labeled LoveDA validation set. Class-wise values denote IoU scores, and bold values indicate the results of CoMS-UNet.

Method	Backbone	Bkg.	Build.	Road	Water	Barr.	Forest	Agri.	mIoU
UNetFormer [22]	R18	54.12	59.58	51.76	63.50	33.35	43.97	50.20	50.93
RS3Mamba [24]	R18-Mamba	50.67	59.29	51.97	52.71	28.26	42.27	50.69	47.98
Segmenter [21]	ViT-T	51.83	55.58	49.95	71.06	24.12	39.62	59.73	50.27
ABCNet [19]	R18	51.97	59.26	52.54	62.97	30.39	37.22	45.82	48.59
CM-UNet [13]	R18	53.31	62.86	53.42	64.28	34.40	41.48	50.51	51.47
**CoMS-UNet (Ours)**	R18	**54.21**	**63.79**	**53.16**	**65.85**	**34.77**	**44.20**	**51.35**	**52.48**

**Table 3:** Ablation study (%) on the ISPRS Vaihingen dataset. Results are reported as the mean ± standard deviation over three independent runs using random seeds 0, 1, and 42. Bold values indicate the best result for each evaluation metric.

MSRM	SAM	CSSMBlock	mF1	OA	mIoU
×	×	×	91.58±0.24	94.50±0.16	84.88±0.41
✓	×	×	92.21±0.10	94.86±0.14	85.93±0.19
×	✓	×	91.95±0.37	94.69±0.20	85.49±0.60
×	×	✓	91.81±0.24	94.62±0.13	85.26±0.40
✓	✓	×	92.29±0.07	94.82±0.08	86.05±0.12
✓	×	✓	92.15±0.02	94.76±0.01	85.84±0.02
×	✓	✓	91.93±0.19	94.65±0.06	85.48±0.31
✓	✓	✓	92.33±0.22	94.83±0.16	86.11±0.35

**Table 4:** Computational complexity and parameter analysis. The mIoU values correspond to the runs using random seed 42, whereas FLOPs and parameter counts are independent of the random seed.

Model	FLOPs@512 ↓	FLOPs@1024 ↓	Param. ↓	mIoU (%) ↑
ABCNet [19]	15.63	62.50	13.39	80.90
UNetFormer [22]	11.74	46.97	11.69	82.77
FTUNetFormer [22]	126.30	499.19	96.14	84.14
CMTFNet [23]	33.07	131.87	30.07	81.86
CM-UNet [13]	12.02	48.08	12.89	85.28
**CoMS-UNet (Ours)**	**38.48**	**153.91**	**30.86**	**86.51**

FLOPs, Param., and mIoU denote floating-point operations, parameter count, and mean Intersection over Union, respectively. FLOPs are reported for a single RGB image at two resolutions: 1×3×512×512, corresponding to the training crop size, and 1×3×1024×1024, corresponding to the full-resolution inference tile. The reported FLOPs measure a single forward pass without test-time augmentation. The mIoU values are evaluated on the ISPRS Vaihingen dataset. Bold values indicate the results of CoMS-UNet.

## Data Availability

The ISPRS Vaihingen dataset can be accessed via the official ISPRS benchmark repository (https://www.isprs.org/resources/datasets/benchmarks/UrbanSemLab/, accessed on 15 June 2026). The LoveDA dataset is available in the Zenodo repository (https://zenodo.org/record/5706578, accessed on 15 June 2026).

## Abbreviations

The following abbreviations are used in this manuscript:

CoMS-UNet	Cohesive Multi-Scale U-Net
MSRM	Multi-Scale Semantic Reorganization Module
SAM	Scale-Aware Module
CSSMBlock	Channel-Spatial Shuffle Mamba Block
ECSBlock	Enhanced Channel-Shuffle Block
LCSBlock	Local Channel-Shuffle Block
DSC	Depthwise Separable Convolution
SS2D	Two-Dimensional Selective Scan
MSAA	Multi-Scale Attention Aggregation
GAP	Global Average Pooling
BN	Batch Normalization
LN	Layer Normalization
GConv	Grouped Convolution
DWConv	Depthwise Convolution
C-Shuffle	Channel Shuffle
C-Attn	Channel Attention
S-Attn	Spatial Attention
CS-Attn	Channel-Spatial Attention
FLOPs	Floating-Point Operations
RGB	Red–Green–Blue
